# Role of PPAR***γ*** in the Differentiation and Function of Neurons

**DOI:** 10.1155/2014/768594

**Published:** 2014-08-26

**Authors:** Rodrigo A. Quintanilla, Elias Utreras, Fabián A. Cabezas-Opazo

**Affiliations:** ^1^Centro de Investigación Biomédica, Universidad Autónoma de Chile, Carlos Antúnez 1920, 750056 Santiago, Chile; ^2^Laboratorio de Neurociencias, Departamento de Neurología, Escuela de Medicina, Pontificia Universidad Católica de Chile, 8330024 Santiago, Chile; ^3^Laboratorio de Dinámica Celular y Neuronal, Departamento de Biología, Facultad de Ciencias, Universidad de Chile, Ñuñoa, 7800003 Santiago, Chile

## Abstract

Neuronal processes (neurites and axons) have an important role in brain cells communication and, generally, they are damaged in neurodegenerative diseases. Recent evidence has showed that the activation of PPAR*γ* pathway promoted neuronal differentiation and axon polarity. In addition, activation of PPAR*γ* using thiazolidinediones (TZDs) prevented neurodegeneration by reducing neuronal death, improving mitochondrial function, and decreasing neuroinflammation in neuropathic pain. In this review, we will discuss important evidence that supports a possible role of PPAR*γ* in neuronal development, improvement of neuronal health, and pain signaling. Therefore, activation of PPAR*γ* is a potential target with therapeutic applications against neurodegenerative disorders, brain injury, and pain regulation.

## 1. Introduction

### 1.1. Peroxisome Proliferator Activated Receptors

Peroxisome proliferator activated receptors (PPARs) are nuclear receptors that induce signaling and transcription of different pathways [1]. Generally, they participate in the regulation of lipids metabolism and glucose homeostasis, and they also are activated by specific ligands [1–3]. The family of PPARs is mostly composed of three known isoforms: PPARα, PPAR*β*/*δ*, and PPAR*γ*. These receptors share a structural homology that consists of four functional units (A, B, C, and D) [1–3]. Unit A/B, located in N-terminal region of the receptor, controls the activation domain by AF-1 ligand, and Units C and D present a DNA binding domain that includes two zinc fingers motives and a docking domain [1–3]. The C-terminal region contains a specific binding domain and a transactivation domain for AF-2 [2]. This region is very important for nuclear localization of the PPARs and other interactions with activator proteins [1–3].

**Figure 1:**
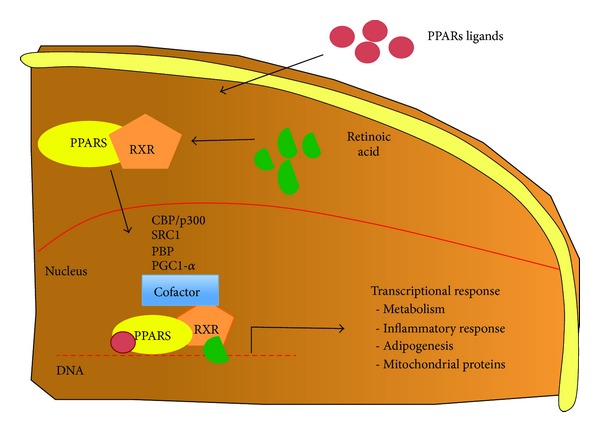
*Overview of PPARs signaling and activation*. The figure shows how PPARs are activated. PPARs ligand enters the cell where it binds to promote receptor dimerization with receptor of 9-cis-retinoic acid (RXR). This complex migrates to the nucleus where it binds to DNA and to different cofactors proteins (CBP/p300, SRC1, PBP, and PGC1-α), to induce the expression of several genes involved in metabolism, inflammatory response, and antioxidant defense.

The binding of specific agonists activates the PPARs response, forming a heterodimer complex between PPARs and retinoic acid receptor (RXR), and then this complex will bind to specific PPRE regions in the DNA to activate different target genes [4]. In addition, this dimer can interact with other coactivators proteins like CBP/p300, SRC1, PBP, and PGC-1α to induce a specific gene expression (Figure 1) [3, 4].

PPARα expression is abundant in liver, kidney, and heart and commonly is present in tissues with high metabolic rate [1, 4]. PPARα is activated by polyunsaturated fatty acids, like docosahexaenoic acid (DHA) and icosapentaenoic acid (EPA), and by fibrate drugs like gemfibrozil and fenofibrate, which are currently used as a treatment for dyslipidemia, metabolic syndrome, and cardiovascular damage [1, 3, 4].

PPAR*β* expression is ubiquitous and their abundance depends on the tissue [5]. To this date, evidence suggests that PPAR*β* is activated like PPARα and apparently plays a role in embryo development [5].

PPAR*γ* is expressed principally in fatty and vascular tissue [6, 7]; however, it has showed their presence in heart and brain tissue, where their activation reduced cardiovascular damage and neurodegeneration [6, 7]. PPAR*γ* is activated by natural ligands like linoleic acid (9- and 13-HODE) and by prostaglandin derivative, 15-deoxi-Δ^12,14^-prostaglandin J_2_ (15d-PGJ_2_), which regulates inflammatory response [3]. Clinical importance of PPAR*γ* has risen especially after the use of TZDs drugs for the treatment of diabetes mellitus type 2 [8, 9]. Treatment with TZDs activates PPAR*γ* pathway reducing insulin resistance and blood glucose levels in patients with diabetes type 2 [9].

Interestingly, the activation of PPAR*γ* receptors by TZDs prevented neurodegeneration and promoted neuronal development in primary neuronal cultures [10].

## 2. PPAR*γ* Activation Prevents Neurodegeneration

In this review, we will discuss evidence that suggests an important role of PPAR*γ* in brain function, neuronal development, and pain signaling. For the studies that cover neuroprotective effects of PPAR*γ*, we will discuss evidence produced in different study models (animal, cells, and patients), which supports the use of PPAR*γ* activation against neurodegeneration. For the effects of PPAR*γ* promoting neuronal development, we will describe evidence obtained from neuronal stem cells (NSCs) and primary neuronal cultures. Finally, as an extension of this review, we will discuss other neurological pathways where PPAR*γ* could be an important player.

### 2.1. Animal Studies

Agonists of PPAR*γ* have been used to reduce neurodegenerative changes in mice models that mimic several neurodegenerative diseases [10]. For example, studies performed in a transgenic mouse that resembles experimental autoimmune encephalomyelitis (EAE) showed that treatment with PPAR*γ* activators delayed neurodegeneration [10]. PPAR*γ* agonists have also showed benefits in experimental models of stroke and ischemia [11–13]. In general, activation of these receptors reduced inflammation and apoptosis and improved memory function [11–14]. Moreover, in a mice model of amyotrophic lateral sclerosis (ALS), a disease that causes paralysis due to loss of motor neurons, treatment with PPAR*γ* agonists like rosiglitazone extends survival and decreases motor neuron loss [15–17].

Beneficial effects of PPAR*γ* activators also have been studied in mice models of Alzheimer's disease (AD). AD is a neurodegenerative disorder that affects a large segment of older population and clinically is characterized by a progressive memory decline of the patient and, later, the presence of brain aggregates of a protein called amyloid-*β* (A*β*) and the accumulation of the hyperphosphorylated form of the tau protein, which later forms intraneuronal aggregates known as neurofibrillary tangles (NFTs) [18]. Some studies explored the possibility that PPAR*γ* activation reduced neuropathological changes in different AD mice models. For instance, PPAR*γ* activation by some TZDs drugs reduced amyloid deposition and reversed cognitive and memory decline in some AD transgenic mice models [18–21]. Treatment with PPAR*γ* agonist rosiglitazone improved hippocampus cognition in the Tg2576 AD mice with no effect on wild type mice [21]. Tg2576 transgenic mouse is an AD study model that presents amyloidosis (accumulation of A*β*), neuronal loss, and cognitive decline [21]. In a different study, oral treatment with PPAR*γ* agonist pioglitazone reduced the A*β* levels within the cortex in the APPswe/PSEN1*δ*E9 (APP/PS1) mice, another mouse model that accumulates A*β* plaques similar to neuropathological features presented in AD [22]. In addition, chronic pioglitazone treatment reduced expression of inflammatory cytokines and enhanced phagocytosis of deposited forms of A*β* [22]. More importantly, reduction in amyloid plaque levels was associated with a reduction of cognitive defects in the drug-treated APP/PS1 mice [22]. Further studies in Tg2576 AD mice extend the use of PPAR*γ* activators against neurodegeneration [23]. Tg2576 mice showed significant impairment in memory and cognition compared with wild type mice [23]. Tg2576 AD mice treated with rosiglitazone improved neuronal function indicated by an increase in neuronal activity [23]. These effects were correlated with an increase in the expression of presynaptic proteins that are reduced in patients with AD [23].

In AD, the inflammatory response is exacerbated in glia and astrocytes and accumulative evidence indicates that neuroinflammation contributes to neuronal dysfunction [18]. Interestingly, Heneka et al. studied if PPAR*γ* activation reduces the expression of proinflammatory cytokines, in order to improve neuronal injury observed in AD [18]. Acute treatment of 10-month-old APPV717I mice with pioglitazone significantly decreased the number of activated microglia and astrocytes in hippocampus and cortex [18]. In addition, pioglitazone treatment reduced the expression of the proinflammatory enzymes like cyclooxygenase 2 (COX2) and inducible nitric oxide synthase (iNOS). Finally, pioglitazone treatment reduced amyloid deposits in the hippocampus and cortex [18]. Complementary studies by Yamanaka et al., using the PPAR*γ* agonist pioglitazone and a novel selective PPAR*γ* modulator, DSP-8658, observed an increase in microglial activation and phagocytosis in the APPV717I mice [24]. PPAR*γ* activators increased A*β* phagocytosis through the upregulation of scavenger receptor CD36 [24]. Furthermore, DSP-8658-treated mice showed improvement in spatial memory performance in APPV717I mice [24].

Further studies explored the role of PPAR*γ* on cyclin-dependent kinase 5 (Cdk5) pathway. Cdk5 is a kinase that apparently plays an important role in neurogenesis and its deregulation is involved in the pathogenesis of AD [25]. Interestingly, effects of early lethality, astrogliosis, and increased neuroinflammation were observed in Cdk5 conditional knock-out mice [25]. More importantly, these effects were significantly reduced with the pioglitazone treatment [26].

Despite large evidence discussed above where PPAR*γ* activation ameliorates neurodegenerative effects in AD and other neurological diseases, some studies had showed opposite results. For example, a recent study published by Dumont et al. explored the effects of peroxisome proliferator-activated receptor coactivator-α (PGC-1α) expression in the Tg19959 mice, another AD mice model that has increased A*β* levels and memory deficits [27]. Binding of PGC-1α with PPAR*γ* induces the expression of different proteins involved in the regulation of mitochondrial biogenesis [28]. Other studies in Huntington disease (HD) showed a significant reduction in mRNA PGC-1α levels, the event that could contribute to the pathogenesis of this disease [28]. Surprisingly, these studies showed that the crossing of the Tg19959 mice with a mouse that overexpresses human PGC-1α exacerbated amyloid and tau pathology [27]. AD-like pathology was accompanied by mitochondrial dysfunction, neuronal death, and an exacerbated hyperactivity in the Tg19959/PGC1-α mice [27].

Beneficial effects of PPAR*γ* had been investigated in other neurological conditions. The stroke is a devastating disease with limited treatment options. In this context, several groups have explored the use of PPAR*γ* activators against neuronal injury [29]. For example, treatment with PPAR*γ* agonists reduced injury and inflammation in a rat model of transient cerebral ischemia [29]. Complementary studies examined PPAR*γ* expression, DNA binding, and PPAR*γ* transcriptional activity after stroke induced in rats [30]. PPAR*γ* expression was dramatically increased in ischemic neurons and the treatment with T0070907, a PPAR*γ* antagonist, reversed rosiglitazone-mediated protection after stroke [29].

Beneficial effects of PPAR*γ* activators were investigated in mice models subjected to damage for ischemia [29–31]. In response to ischemia, expression of PPAR*γ* gene was significantly increased in neurons, suggesting that neuronal PPAR*γ* may be a primary target for PPAR*γ*-agonist-mediated neuroprotection [30, 31]. In other studies, Zhao et al. evaluated the contribution of PPAR*γ* to ischemic injury, generating conditional neuron-specific PPAR*γ* knock-out mice (PPAR*γ*-KO) [32]. PPAR*γ* deficiency caused increased brain damage and oxidative stress in response to cerebral artery occlusion [32]. Primary cortical neurons from PPAR*γ*-KO mice showed increased neuronal death, reduced expression of SOD1 (superoxide dismutase 1), catalase, glutathione S-transferase, and uncoupling protein-1 (UCP-1) after ischemia [32], suggesting that PPAR*γ* is an important factor in the regulation of the antioxidant response in the brain.

PPAR*γ* effects were also investigated in animals submitted to spinal cord injury (SCI) [33]. Compared with the control groups, rosiglitazone treatment significantly increased locomotor recovery, reduced NF-*κ*B expression, and increased the proliferation of endogenous neuronal precursors cells (NPCs) in animals subjected to spinal injury [33].

Finally, the protective role of a natural PPAR*γ* ligand and 15-deoxy-delta12,14-prostaglandin J_2_ (15-PGJ_2_) in ischemia-reperfusion has been reported [34]. The treatment with 15d-PGJ_2_ decreased expression of autophagic proteins (LC3-II, Beclin 1, cathepsin-B, and LAMP1) in ischemic cortex of animals with artery occlusion, exerting neuroprotection through the inhibition of neuronal autophagy [34].

### 2.2. Neuronal Cells Studies

As we described before, PPAR*γ* has been proposed as a therapeutic target against neurodegenerative diseases because of its anti-inflammatory action in glial cells [18, 22]. However, several reports indicate that PPAR*γ* agonists induce neuroprotective actions through an independent pathway [35, 36]. For example, Fuenzalida et al. showed that the rosiglitazone protected hippocampal neurons against A*β* toxicity and apoptosis induced by nerve growth factor (NGF) deprivation [35]. The protective effects of rosiglitazone were associated with an increase in the expression of Bcl-2, an antiapoptotic protein [35]. Interestingly, PC12 cells expressing a dominant negative mutant of PPAR*γ* showed an enhanced sensitivity to neurotoxic changes induced by A*β*, including apoptosis, neurites damage, oxidative stress, and mitochondrial injury [35]. In the same context, our group explored the effects of PPAR*γ* activators on hippocampal neurons treated with A*β* [36]. These studies showed that activation of PPAR*γ* by troglitazone and rosiglitazone protects hippocampal neurons against A*β*-induced neurodegeneration [36]. In addition, PPAR*γ* activation results in the modulation of Wnt signaling components, including the inhibition of glycogen synthase kinase-3*β* (GSK-3*β*) and an increase of the *β*-catenin levels [20, 36]. GSK-3*β* is a kinase that has been suggested to be responsible for the anomalous tau hyperphosphorylation in AD [37], and the activation of Wnt pathway by proper Wnt ligands protected hippocampal neurons and AD mice exposed to A*β* [38].

Also, protective effects of PPARs activators on neuronal cells have been related with an increase in the antioxidant response [16, 17, 39]. For instance, Santos et al. showed that an increase in peroxisomal proliferation attenuated A*β* toxicity in hippocampal neurons [39]. Pretreatment with Wy-14.463 (Wy), a peroxisome proliferator and a PPARα activator, prevented neuronal death and neurites loss induced by the A*β* [39]. Moreover, neurons treated with this compound showed an increase in the number of peroxisomes, with a concomitant increase in catalase activity, reducing the production of intracellular reactive oxygen species (ROS), and prevented mitochondrial dysfunction in neurons exposed to both H_2_O_2_ and A*β* [39].

PPAR*γ* agonists have also been tested in neuronal cells treated with acetaldehyde, a toxin that mimics Parkinson disease (PD) neurodegeneration [40]. Acetaldehyde is an inhibitor of mitochondrial function and induced oxidative stress and apoptosis in neuronal cells [41]. In these studies, the apoptosis induced by acetaldehyde was moderately reversed by rosiglitazone treatment in human neuroblastoma SH-SY5Y cells [41]. In addition, the treatment with rosiglitazone induced the expression of antioxidant proteins like Bcl-2 and Bax [41]. Complementary studies examined the role of PPAR*γ* activation against PD in neuronal cells treated with 1-methyl-4-phenylpyridinium ion (MPP^+^). MPP is an inhibitor of mitochondrial complex I that has been widely used as a neurotoxin that mimics PD-like syndrome [42]. Human neuroblastoma SH-SY5Y cells treated with both MPP^+^ and rosiglitazone showed a reduction of apoptosis and an increase in the expression of superoxide dismutase (SOD) and catalase [42].

Important evidence indicates that PPAR*γ* activators can ameliorate neurodegeneration in HD [28, 43–45]. HD is a neurodegenerative disease caused by the pathological elongation of CAG repeats in exon 1 of the huntingtin protein gene and is characterized by dysfunction and loss of striatal and cortical neurons [44]. Accumulative evidence suggests that mitochondrial impairment could be part of neuropathological mechanisms behind HD [28, 44]. In this context, previous findings of our group studied the potential neuroprotective role of PPAR*γ* activation on preventing the loss of mitochondrial function in HD [43, 44]. PPAR*γ* activation by rosiglitazone prevented the mitochondrial failure and reduced oxidative stress that occurred when striatal cells that express mutant huntingtin were challenged with calcium stress [43].

### 2.3. Clinical Studies

Positive results of PPAR*γ* activators against neurodegeneration in cell lines and animal models have encouraged testing these compounds in patients affected by neurodegenerative disorders [18–21]. For instance, the effects of the PPAR*γ* agonist pioglitazone on cognition, cerebral blood flow (CBF), and plasma levels of A*β* were tested in a controlled trial in patients with mild AD during 6 months [29]. Patient group treated with pioglitazone improved cognition and CBF, while untreated group showed no improvement [29]. The plasma A*β*40/A*β*42 ratio increased in the control group but showed no significant changes in the pioglitazone group [29]. In another study, authors evaluated the effects of rosiglitazone on cognition and plasma levels of A*β* in AD patients [30]. Patients with AD that received rosiglitazone exhibited an improvement in memory (at the 4th and 6th months) and selective attention (the 6th month) compared to untreated patients [30]. Plasma A*β* levels were unchanged in subjects treated with rosiglitazone but decreased for untreated subjects [30].

In addition, in a case report, Sundararajan et al. investigated the therapeutic potential of pioglitazone in a patient with secondary progressive multiple sclerosis (MS) [31]. Treatment with pioglitazone attenuated her cognitive decline and improved fine coordination, and after 3 years of treatment the patient continued being clinically stable, with no adverse events [31]. Also, in a randomized controlled trial of 5238 patients with diabetes type 2 who had evidence of macrovascular disease, treatment with pioglitazone for 34 months reduced the combined risk of heart attacks, strokes, and death by 16% in high-risk patients [32]. In a different study, 30 stroke patients received treatment with pioglitazone or rosiglitazone for 36 days after accident [33]. Treatment with PPAR*γ* agonists showed a significant improvement in functional independence measure (FIMTM), indicating that the administration of TZDs drugs improved their functional recovery by the modulation of the neuroinflammatory response following stroke [33].

### 2.4. The Unsuccessful Use of PPAR*γ* Activators against Neurodegeneration

Despite all evidence that suggests PPARs activators prevented or delayed neurodegenerative changes, several studies delivered conflict results [29, 45–47]. For instance, in phase III trial studies, memory cognition was not significantly improved in AD patients treated with PPAR*γ* activators [47]. This evidence suggests that perhaps the mechanism of the action of PPAR*γ* agonists in animal models of amyloid deposition may differ from those in humans [48]. Other studies showed no evidence of improvement in cognition and functional tasks, in AD patients treated with rosiglitazone [47, 48], and in AD patients positive for apolipoprotein *ε*4 allele, the treatment with rosiglitazone showed a significant decline in cognition [48]. Complementary studies in ALS showed that, in a phase II clinical trial, the treatment with pioglitazone had no positive effects on the survival of ALS patients treated with riluzole (a drug that extends lifespan of ALS patients) [49].

In addition, studies using different neuronal cell models showed no benefit of PPAR*γ* activation against neurodegeneration [50]. For instance, studies that explored positive effects of troglitazone and pioglitazone against ALS showed that treatment with both drugs did not promote the survival of hippocampal neurons and rat motoneurons [50]. Also, it has reported that treatment with 15d-PGJ_2_ induced neurite degeneration and nuclear fragmentation, in primary neurons and SH-SY5Y neuroblastoma cells [51]. Moreover, the combined treatment with both ciglitazone (another PPAR*γ* agonist) and 15d-PGJ_2_ generated neurotoxicity in cultured cerebellar granule neurons, in a dose response manner [52].

Finally, secondary effects of PPAR*γ* activators have been reported in several neurodegenerative diseases [53]. For example, in Friedreich's ataxia the use of TZDs drugs caused a decrease in the number of fast fibers and an increase in mitochondrial biogenesis in cardiac muscle, enhancing the incidence of heart failure and thrombosis in these patients [53]. In addition, the use of pioglitazone in nondiabetic patients with AD showed a 28.6% of increase in peripheral edema compared to patients treated with placebo [54].

## 3. The Role of PPAR***γ*** in Neuronal Development

### 3.1. PPAR*γ* and Neuronal Stem Cells

Recent evidence suggests that PPAR*γ* could have a potential role in neuronal development [55]. In physiological conditions PPAR*γ* expression was found in embryo mouse brain and in neuronal stem cells (NSCs) [55]. In contrast, extremely low levels of PPAR*γ* were observed in adult mouse brain [55]. More important, PPAR*γ* agonists promoted oligodendrocyte differentiation of mouse NSCs, by modulating expression of differentiation genes [56]. Moreover, activation of PPAR*γ* induced expression of neurogenic differentiation factor (Neurod1), a member of the basic helix-loop-helix (bHLH) transcriptional factor that plays a role in the development of nervous and endocrine systems [56]. These studies in Neurod1-null mice exhibited behavioral abnormalities due to a reduction in the number of sensory neurons [56]. Thus, the upregulation of selective differentiation factors could be a mechanism by which PPAR*γ* agonists promote differentiation of NSCs.

It has been suggested that activation of PPAR*γ* could be an interesting therapeutic target against AD [17, 19]. In this context, Cannabidiol (CBD), a Cannabis derivative, has attracted much attention because of its promising neuroprotective properties [57]. New studies suggest that neuroprotective effects of CBD could be mediated through PPAR*γ* pathway [57]. Interestingly, due to its interaction with PPAR*γ*, CBD was able to stimulate hippocampal neurogenesis [57], indicating that CBD may exert protective functions through an increase of neuronal population by the activation of PPAR*γ* [57].

### 3.2. PPAR*γ* Activation Induces Neuronal Differentiation

An important role of PPAR*γ* in the differentiation of neuronal cells has been demonstrated in different studies [58, 59]. PPAR*γ* is expressed in the central nervous system [30, 36], and 15d-PGJ_2_, a natural PPAR*γ* ligand, stimulated neurite outgrowth in pheochromocytoma 12 (PC12) cells stimulated with NGF [60]. In addition, we reported that PPAR*γ* is present in rat hippocampal neurons and that its activation by TZDs prevented axon degeneration, neurite loss, and mitochondrial impairment induced by A*β* [35, 36]. Importantly, the treatment with troglitazone induced an increase in axon length and neurite outgrowth compared with untreated neurons [36, 61].

In addition, the role of PPAR*γ* in neuronal development has been studied in neuronal cells treated with retinoic acid (RA) [62]. RA regulates gene expression by activating the nuclear retinoic acid receptor (RXR) inducing neuronal outgrowth in neuroblastoma cells [63]. Activation of RA induced differentiation of stem cells to neuronal progenitors through activation of FABP5/PPAR*γ* pathway [61].

Complementary to studies in which PPAR*γ* activation induces neuronal differentiation, recent evidence suggests an important role of PPAR*γ* in neuronal polarity [61]. Studies made on hippocampal neurons indicated that activation of PPAR*γ* by TZDs drugs enhanced axonal growth [61]. This effect on axonal growth was accompanied by an increase in PPAR*γ* expression and was completely prevented by the use of GW9662, a specific PPAR*γ* antagonist [61]. The enhanced axonal growth induced by PPAR*γ* activators was prevented by SP 600125, an inhibitor of c-Jun N-terminal kinase (JNK), indicating that the effect of PPAR*γ* on neuronal polarity was through the activation of JNK pathway [61].

Finally, the effects of PPAR*γ* activation have been also studied in AD mice model that expressed apolipoprotein (Apo-E4) [64]. Apo-E4 is a major genetic risk factor for AD and exerts neuropathological effects through multiple pathways, including reduction of dendritic spine density and mitochondrial dysfunction [65]. Apo-E4 fragments are neurotoxic and cause neurodegeneration and behavioral deficits in transgenic mice [66]. In this context, Brodbeck et al. studied the effects of rosiglitazone on dendritic spine density in AD mice that expressed Apo-E4 [64]. Treatment with rosiglitazone significantly increased dendritic spine density in a dose-dependent manner in cultured cortical neurons from wild type mice [64]. This effect was prevented by GW9662, suggesting that rosiglitazone exerts this effect by activating the PPAR*γ* [64]. Furthermore, dendritic spine density was significantly decreased in cortical cultures obtained from AD Apo-E4 mice, and treatment with rosiglitazone rescued this detrimental effect [64].

## 4. The Role of PPAR***γ*** in Pain Signaling

Several studies using experimental models have showed that administration of PPAR ligands reduces inflammation, suggesting their possible use for treating human inflammatory and neuropathic pain [67]. Earlier in 1995, a prominent expression of PPARs in the thalamus was reported, particularly in the posterior part of the ventral medial nucleus, a site responsive to pain and cold stress, suggesting the possibility that PPARs might play a role in modulating response to thermal and pain sensations [68].

Further studies reported that the treatment with TZDs drugs, such as rosiglitazone and pioglitazone, prevented myelin loss, reduced neuropathic pain, and improved motor function recovery after spinal cord injury [69]. In addition, Ajulemic acid, which has a potent analgesic and anti-inflammatory activity, directly interacts with PPAR*γ* suggesting that this may be a pharmacologically relevant receptor for this compound and a potential target for drug development in the treatment of pain [70]. In addition, new studies have suggested PPAR*γ* as a new target for treating chronic pain [71, 72]. Thus, the expression and function of PPAR*γ* in spinal cord were reported [71]. Moreover, intrathecal administration of rosiglitazone reduced allodynia (increased sensitivity to pain from a stimulus that normally does not provoke pain) and hyperalgesia (increased sensitivity to pain from a stimulus that normally provokes pain) in the spared nerve injury (SNI) mouse model of neuropathic pain [71]. These studies suggest that new or current drugs that targeted spinal PPAR*γ* may yield important therapeutic effects for neuropathic pain [71]. Also it was reported that pioglitazone administration reduced tactile allodynia and thermal hyperalgesia in partial sciatic nerve ligation (PSL), a study model for neuropathic pain [73]. PSL-induced upregulation of TNFα and IL-6 was suppressed by pioglitazone treatment, indicating that pioglitazone alleviates neuropathic pain through attenuation of proinflammatory cytokine upregulation by PPAR*γ* [73]. Also, systemic administration of TZDs reduces peripheral inflammation in vivo suggesting that pharmacological activation of PPAR*γ* in the brain rapidly inhibits local edema and the spinal transmission of noxious inflammatory signals [74]. Interestingly, it was showed that PPAR*γ* is crucial for coupling ibuprofen to RhoA inhibition and subsequently induces neurite growth in neurons, providing additional therapeutic targets to the disorders characterized by RhoA activation, including spinal cord injury and AD [75]. It was also reported that rosiglitazone attenuates postincisional pain by regulating macrophage polarization [76] and alleviated the development of inflammatory pain, possibly through regulating macrophage infiltration [77]. These observations suggest that PPAR*γ* signaling in macrophages may be a potential therapeutic target for the treatment of acute pain development [77]. Finally, oral or intraperitoneal administration of pioglitazone prevents multiple behavior sings of somatosensory hypersensitivity [72]. Thus, pioglitazone reduces spinal glial and stimulus-evoked p-ERK activation and reduced neuropathic pain [72].

## 5. Conclusions

**Figure 2:**
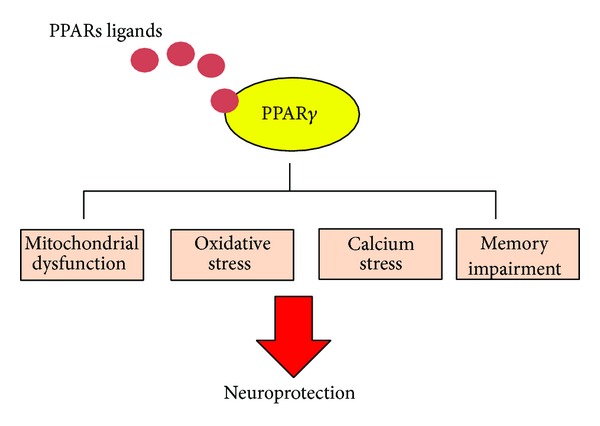
*Activation of PPAR* *γ* *improves neuronal health*. Several neurodegenerative diseases showed clear deficiencies in mitochondrial function, oxidative stress, and memory impairment. Activation of PPAR*γ* by natural ligands or TZDs could prevent these neurodegenerative changes mainly improving mitochondrial function and increasing antioxidant capacity in neurons.

Evidence discussed here clearly shows the importance of PPAR*γ* promoting the development and health of neurons. Accumulative evidence suggests that PPAR*γ* induces neuronal differentiation by a mechanism that implicates activation of PPAR*γ*-dependent transcription and also activation of secondary pathways. Evidence obtained from pharmacological activation of PPAR*γ* by TZDs drugs suggests a possible therapeutic use against neurodegenerative diseases (Figure 2). Concomitantly, studies of PPAR*γ* activation showed important effects against oxidative stress, mitochondrial dysfunction, and apoptosis in several cells models that resemble AD, HD, ALS, and SCI. Also, a large part of this evidence was corroborated in mice models for each of these neurological disorders, and additionally PPAR*γ* activation improved cognitive decline observed in several neurodegenerative diseases. Moreover, the effects of PPAR*γ* ligands on neuroinflammation in animal models suggest their possible use for treating human inflammatory pain and neuropathic pain.

Altogether these observations suggest an important role for PPAR*γ* in maintaining normal function of the brain and preventing neuronal damage induced by stressors and aging.
